# Associations Between Dry Land Strength and Power Measurements with Swimming Performance in Elite Athletes: a Pilot Study

**DOI:** 10.2478/v10078-011-0065-2

**Published:** 2011-10-04

**Authors:** Pedro Morouço, Henrique Neiva, Juan J. González-Badillo, Nuno Garrido, Daniel A. Marinho, Mário C. Marques

**Affiliations:** 1 Department of Sport Sciences, University of Beira Interior (UBI), Covilhã, Portugal; 2 Research Centre for Sport Sciences, Health and Human Development (CIDESD), Vila Real, Portugal; 3 Research Center for Human Movement Sciences, Polytechnic Institute of Leiria, Leiria, Portugal; 4 University of Pablo de Olavied, Seville, Spain; 5 Department of Sport Sciences, Exercise and Health, University of Trás-os-Montes and Alto Douro (UTAD), Vila Real, Portugal

**Keywords:** countermovement jump, squat, bench press, lat pull down back, tethered swimming

## Abstract

The main aim of the present study was to analyze the relationships between dry land strength and power measurements with swimming performance. Ten male national level swimmers (age: 14.9 ± 0.74 years, body mass: 60.0 ± 6.26 kg, height: 171.9 ± 6.26, 100 m long course front crawl performance: 59.9 ± 1.87 s) volunteered as subjects. Height and Work were estimated for CMJ. Mean power in the propulsive phase was assessed for squat, bench press (concentric phase) and lat pull down back. Mean force production was evaluated through 30 s maximal effort tethered swimming in front crawl using whole body, arms only and legs only. Swimming velocity was calculated from a maximal bout of 50 m front crawl. Height of CMJ did not correlate with any of the studied variables. There were positive and moderate-strong associations between the work during CMJ and mean propulsive power in squat with tethered forces during whole body and legs only swimming. Mean propulsive power of bench press and lat pull down presented positive and moderate-strong relationships with mean force production in whole body and arms only. Swimming performance is related with mean power of lat pull down back. So, lat pull down back is the most related dry land test with swimming performance; bench press with force production in water arms only; and work during CMJ with tethered forces legs only.

## Introduction

Strength parameters have been recently proposed as one of the multi-factorial phenomenon that enhances swimming performance (Tanaka et al., 1993; Barbosa et al., 2010). Nevertheless, the assessment of specific muscle power output of both arms and legs seems to be underlying in swimming (Swaine et al., 2010) as the locomotion in the aquatic environment is highly complex, being difficult to assess the magnitude of these forces (Morouço et al., 2011). It has been purposed that as the distance diminishes strength role increases, when comparing with technical parameters (Wilke and Madsen, 1990; Swaine, 2000; Stager and Coyle, 2005; Morouço et al., 2011). Unfortunately, results trying to support this idea remain inconclusive (Girold et al., 2007; Aspenes et al., 2009; Garrido et al., 2010), and more studies are necessary to clarify the specificity of the strength training methods in swimmers.

Tethered swimming was proposed as a methodology to evaluate the force a swimmer can exert in water (Magel, 1970). In fact, several approaches have shown its proximity with swimming performance in short distance events (Yeater et al., 1981; Costill et al., 1986; Christensen and Smith, 1987; Keskinen et al., 1989; Fomitchenko, 1999; Dopsaj et al., 2003; Kjendlie and Thorsvald, 2006; Morouço et al., 2011). These findings suggest that tethered swimming might be a useful, not expensive, not invasive, small time consuming methodology to evaluate one major factor (strength) influential of sprint swimming performance; even recognizing that the movements relative to the water are somehow different than in a free swimming situation (Adams et al., 1983; Maglisho and Maglisho, 1984).

There have been several studies successfully relating the anaerobic power in dry land with swimming velocity in front crawl (Sharp et al., 1982; Hopper et al., 1983; Hawley et al., 1992; Johnson et al., 1993). Yet, the relationship between power output in dry land exercises, apart from isokinetic methods, remains unanswered. Actually, strength and power assessment may be useful to understand the importance of power output for swimming performance, and moreover to improve training programs. This is well stated as the movement velocity with different loads is frequently disregarded in the practice of strength training (Badillo and Medina, 2010). Garrido et al. (2010) evaluated 28 young competitive swimmers aiming to identify which dry land strength and power tests were better associated with sprint swimming performance. These authors presented moderate but significant relationships between strength/power variables with 25 and 50 m sprint tests (0.542 < ρ < 0.744; p < 0.01). These results are in accordance with previous published of Strzala and Tyka (2009) that evaluated average power produced by arms and legs in a dry land ergometer. In fact, higher correlations were reported between power and shorter distance swam (25 m vs. 100 m). However, the specificity of leg movements in order to produce propulsion in water seems quite different from the movements used in cycle ergometer (Swaine et al., 2010). Therefore, this higher correlation in shorter distances may be explained by the push of the wall in the start and the turning benefit (Keskinen et al., 2007). Thus, complementary studies relating these parameters with force production in water by the lower limbs are required.

To the best of our knowledge, few studies examined the relationships between dry land exercises parameters with tethered forces and swimming performance. Here, only Crowe et al. (1999) related different strength and power parameters with swimming performance and tethered forces. However, these authors studied a heterogeneous sample, with subjects of different swimming and strength abilities, analyzing men and women. Therefore, the main aim of the present study was to identify what type of dry land tests are better associated with tethered forces and short distance swimming performance. It was hypothesized that variables obtained through countermovement jump, squat, bench press, and lat pull down back, would significantly correlate with tethered swimming force production and short distance swimming performance.

## Material and Methods

### Subjects

Ten male national level swimmers (age: 14.9 ± 0.74 years, body mass: 60.0 ± 6.26 kg, height: 171.9 ± 6.26, 100 m long course front crawl performance: 59.9 ± 1.87 s) participating on regular basis in regional and national level competitions volunteered as subjects. Parents and coaches gave their consent for the swimmers participation in this study. All procedures were in accordance to the Declaration of Helsinki in respect to Human research. The Ethics Committee of the hosting University approved the study design. Body mass was assessed through a bioelectric impedance analysis method (Tanita BC 420S MA, Japan). Performance index was assessed through personal best time in 100 m freestyle long course swimming competitions, within 2 months prior to data collection.

### In water tests

All tests were performed in a 50 m indoor swimming pool (27.5°C of water temperature) during the competitive period of the spring training cycle. In day one, after a 1000 m low intensity warm-up, each subject performed three repetitions of 30 s maximum front crawl tethered swimming: first using whole-body; second with arms only; and third with legs only. A 30 min of active recovery between bouts was controlled. Subjects were wearing a belt attached to a steel cable (sufficiently stiff that its elasticity could be neglected). A detailed description of the measuring device used in this study has recently been reported elsewhere (Morouço et al., 2011). Preceding the data collection, subjects swam 5 s low intensity, using limbs according to repetition. In the second repetition, a fluctuation device placed between the thighs and another swimmer (instructed that legs shouldn’t be pulled), were used to stand up the legs of the swimmer evaluated. For the legs only test, a fluctuation device was used in one hand, while the other hand was kept alongside the body. The end of the test was set through an acoustic signal. In all repetitions, the swimmers were told to follow the breathing pattern they would normally apply during 50 m freestyle event. The subjects were verbally encouraged throughout the tests, enhancing them to maintain maximal effort over the duration of the experiment. In day two, after a 1000 m low intensity warm-up, each subject performed one 50 m maximal front crawl swim with an underwater start.

### Dry land tests

All tests were performed in a gym starting with 5 min of stationary cycling at a self-selected easy pace, 5 min of static stretches and joint mobilization exercises. In day three, using a dynamic measurement system (T-Force System, Ergotech, Murcia, Spain), each participant executed n repetitions (5 min rest) in concentric only bench press. Initial load was set at 10 kg and was gradually increased in 10 or 5 kg increments until mean propulsive velocity (MPV) got lower than 0.6 m.s^−1^. Following a 30 min rest with active recovery, participants replicated the methodology for Squat, until a MVP lower than 0.9 m.s^−1^ was obtained. A detailed description of the measuring device used in this study has recently been reported elsewhere (Medina and Badillo, 2011). A smith machine was used to ensure a smooth vertical displacement of the bar along a fixed pathway. In day four, same equipment was used. Each subject executed n repetitions (5-min rest) in lat pull down back. Initial load was set at 10 kg and was gradually increased in 10, 5 or 2.5 kg increments until MPV got lower than 0.6 m.s^−1^. After a 30 min rest with active recovery, participants carried out 3 maximal countermovement jumps (Ergojump, Globus, Italy), separated by 1-min rests.

### Data analysis

**Figure 1:**
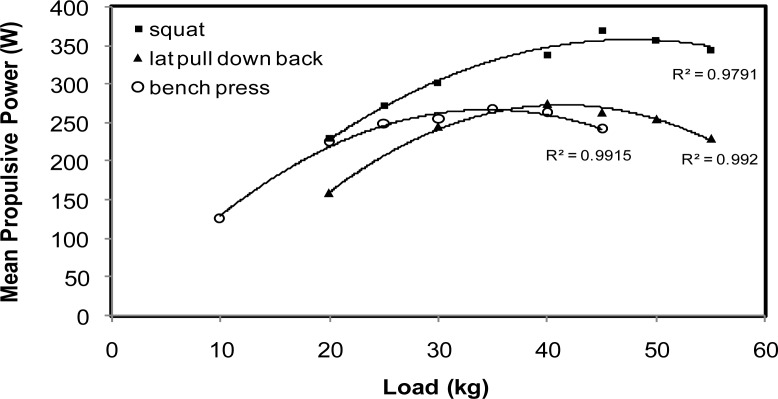
Load-power relationships for one representative subject, for each test.

Individual force to time - F(t) - curves of tethered forces were assessed and registered. As the force vector in the tethered system presented a small angle to the horizontal, computing the horizontal component of force, data was corrected. Average force values during the 30 s test for whole-body (avgFWb); for arms-only (avgFAr); and legs-only (avgFLg) were then calculated. The swimming velocities were estimated according to formula v50 = 50.Δt^−1^; where Δt is the chronometric time in the test. The height of the center of gravity in the countermovement jump (hCMJ) was obtained using the jump fly time. Subsequently, the work was estimated according to formula WCMJ = mgΔh; where m is the body mass (kg), g is the gravitational acceleration (m.s^−2^) and Δh is the elevation of the center of gravity (m). From the dynamic measurement system, data was stored on disk for subsequent analysis. Mean power of the propulsive phase was assessed for each load (cf. figure 1) and maximum value obtained was registered for each test: squat (MPPsq); bench press (MPPbp) and lat pull down back (MPPlpd).

### Statistical analysis

Standard statistical methods were used for the calculation of means and standard deviations (SD) from all dependent variables. The Shapiro-Wilk test was applied to determine the nature of the data distribution. Since the reduce sample size (N < 30) and the rejection of the null hypothesis in the normality assessment, non-parametric procedures were adopted. Spearman correlation coefficients (ρ) were calculated between in water and dry land parameters assessed. Significance was accepted at the p<0.05 level.

## Results

The mean ± SD value for the 50 m sprint test was 1.69 ± 0.04 m.s^−1^. The mean ± SD values of mean force production in tethered swimming tests were 95.16 ± 11.66 N for whole body; 80.33 ± 11.58 N for arms only; and 33.63 ± 7.53 N for legs only. The height assessed in the CMJ was 0.37 ± 0.05 m, being calculated the correspondent work of 219.30 ± 33.16 J. The maximum mean propulsive power in the squat, bench press and lat pull down back were 381.76 ± 49.70 W; 221.77 ± 58.57; and 271.30 ± 47.60 W, respectively. The Table 1 presents the correlation coefficients (ρ) between swimming velocities and average force in tethered tests with dry land variables assessed. It was found significant associations between in water and dry land tests. Concerning the CMJ, work during the jump revealed to be more associated with in water variables, than the height. Both tests that involve the lower limbs musculature (CMJ and squat) presented significant relationship with force production in water with the whole body and legs only, but not with swimming velocity. In bench press and lat pull down back, significant correlations were observed with force production in water with the whole body and arms only, and with swimming velocity for the lat pull down back. Added to that, in the tethered swimming tests, arms only presented a moderate correlation with swimming performance (ρ = 0.68, p = 0.03).

## Discussion

The aim of this study was to analyze the associations between dry land and in water tests. The mean power of the propulsive phase in the lat pull down back was the only parameter that correlated significantly with swimming performance. Additionally, there were significant associations between dry land tests and force exerted in water through tethered swimming.

Concerning in water tests, velocity and mean force in tethered swimming seem to present descriptive data similar to other papers in the literature for the same age and gender (Rohrs and Stager, 1991; Taylor et al., 2003b). As the average force production exerted by the swimmers was assessed in water, values were not related to body mass, as the body weight of the body is reduced to a few kilograms when submersed in water (Taylor et al., 2003a). The relative contribution of arms and legs to tethered forces in front crawl swimming remains uncertain. In fact, Yeater et al. (1981) stated that mean forces with arms only and legs only are significantly lower than the whole stroke force in the whole body swimming. In the present study those differences are also noticeable (p = 0.001 and p = 0.000, respectively), nevertheless with the arms only presenting a higher value than legs only, contradicting the study previous referred. Even so, special attention should be given to the role of the leg kicking (35.34% of the whole body mean value). This data may suggest that a greater proportion of whole body force exerted in water might be done by legs, corroborating the recent findings of Swaine et al. (2010). It is also noticeable that the sum of arms and leg tethered forces (avgFAr + avgFLg) is higher than the whole body forces (avgFWb), but not about the double as referred by Yeater et al. (1981). The reason for this higher sum remains uncertain and more studies are required.

In short activity patterns (e.g. jumping) muscle strength plays a major role, particularly considering its ability to develop it fast (Bencke et al., 2002). In fact, it is assumed that there is a good correlation between lower limb maximum strength and maximum jump height. However, taking into consideration that maximum force does not represent maximum velocity, power developed should be taken into consideration. The CMJ height and work values are somehow similar to referred in literature, according to age and gender. However, there are no values of mean power in the propulsive phase of dry land tests, with which to compare our results. There were obtained higher values in squat, followed by lat pull down and bench press.

Studies have stated the relationship between explosive strength of leg extensor muscles and swimming performance (Keskinen et al., 2007; Strzala et al., 2007; Strzala and Tyka, 2009). Yet, these relationships are pointed to be enhanced by the turning benefit (Keskinen et al., 2007). In the present study, the importance of lower limbs strength was consciously reduced with the underwater start of the 50 m free swimming test, and with a long course pool used. Thus, both hCMJ and WCMJ did not correlate with swimming performance. Still, WCMJ and MPPsq presented a high correlation with force production in tethered swimming with the legs only, and whole body. These associations were expected as the musculature involved in both tests relies mainly in the lower limbs and core.

Johnson et al. (1993) have reported that swimming power (0.84 < r < 0.88), but not dry land measures of strength (r = 0.55) and power (r = 0.74), enhance success in freestyle swimming. However, these authors evaluated one maximum repetition (1RM) bench press which is more related to maximum force than with explosive force (Badillo and Medina, 2010). Also, in that study the swimmer range of age was 14 – 22 years. This seems to be a heterogeneous sample, especially when in this spectrum of ages significant changes in somatotype occur. On the contrary, Garrido et al. (2010) evaluating young competitive swimmers presented a moderate but significant correlation between 1RM bench press and swimming performance (both 25 and 50 m tests; ρ ∼ −0.58; p < 0.01). This incongruous investigations point out that the role of strength and power to force production in water and, consequently to swimming performance, remain uncertain. Simultaneous dry land power, swimming power and swim performance have been previously studied. Crowe et al. (1999), evaluated 1RM in bench press, lat pull down and triceps press. Front crawl tethered swimming 30 s maximal effort was measured and swimming performance was based in 50 m and 100 m distances. In both men and women 1RM in the three strength measures were significantly related with tethered forces. Corroborating this data, in the present study mean propulsive power appears to play an important contribution in the tethered swimming performance (0.65 < ρ <0.75). Both bench press and lat pull down back involve mostly the musculature of the upper body. Therefore, it was expected that power evaluated through these tests would relate with the force produced by arms only in tethered swimming. Indeed, the approach of the present study seems to be more specific as most of the investigations used isokinetic and isometric tests as strength indexes (Marques et al., 2008). Thus, mean propulsive power of the current subjects in bench press and lat pull down back presents a high correlation with tethered forces with arms only (0.69 < ρ < 0.73; p < 0.05), and with whole body. Regarding the swimming performance, only MPPlpd and avgFAr presented significant correlations with velocity. These records seem to be in accordance with Yeater et al. (1981) and Crowe et al. (1999), respectively. Indeed, Crowe et al. (1999) only reported statistical relationship between swimming performance with 1RM lat pull down, and merely in women (r = 0.643, p < 0.05).

To the best of our knowledge, this study was the first to assess the mean power of the propulsive phase in three dry land tests, and to associate this parameter with force production in water and swimming performance. As a conclusion, the present study revealed moderate to high associations between dry land and in water variables. Work during CMJ is a better estimator of force production in water, than height. Squat mean power is related with legs force production in water, and bench press and lat pull down back with arms only tethered forces. Lat pull down back is the most associated dry land test with swimming performance, for the present study.

## Figures and Tables

**Table 1:** Correlation coefficients (ρ) between in water and dry land tests variables

Parameters	**hCMJ**	**WCMJ**	**MPPsq**	**MPPbp**	**MPPlpd**
**avgFWb**	0.10 (p = 0.79)	0.75 (p = 0.01)	0.73 (p = 0.02)	0.65 (p = 0.04)	0.65 (p = 0.04)
**avgFAr**	−0.10 (p = 0.79)	0.27 (p = 0.45)	0.60 (p = 0.07)	0.73 (p = 0.02)	0.69 (p = 0.03)
**avgFLg**	0.17 (p = 0.64)	0.76 (p = 0.01)	0.64 (p = 0.04)	0.40 (p = 0.26)	0.27 (p = 0.45)
**v50**	0.04 (p = 0.92)	0.33 (p = 0.35)	0.36 (p = 0.31)	0.60 (p = 0.07)	0.68 (p = 0.03)
